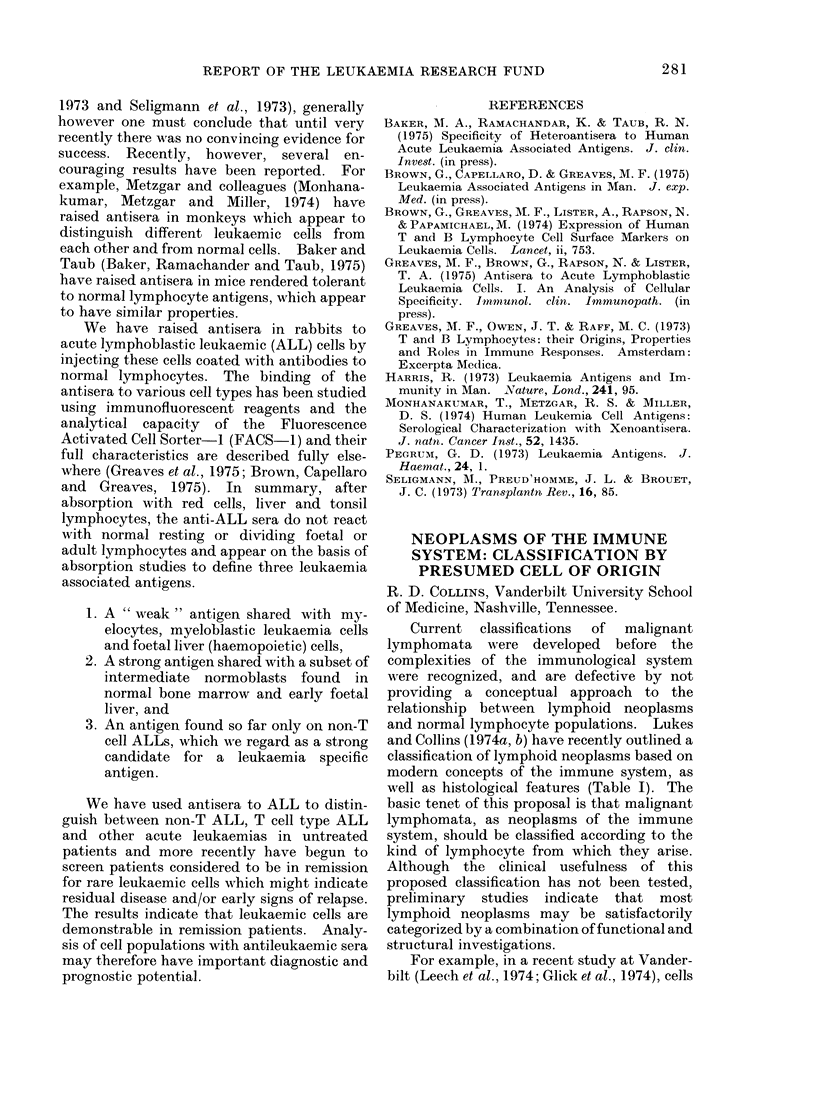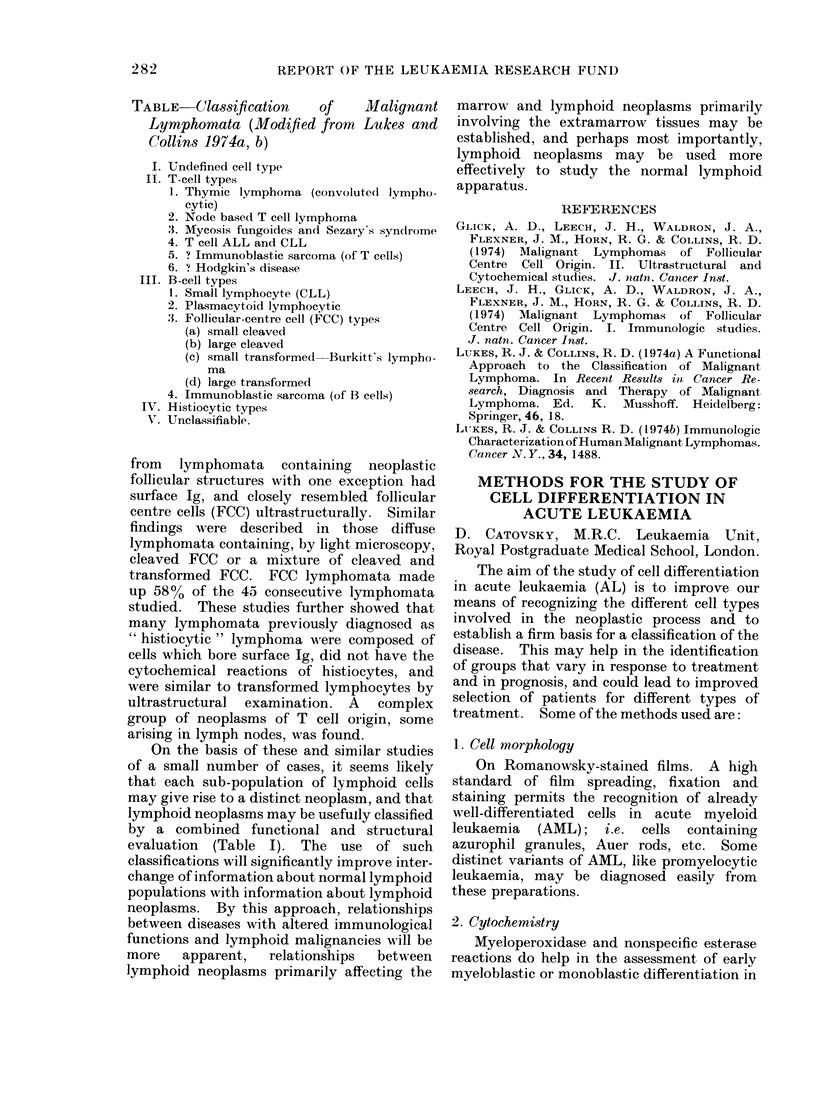# Proceedings: Neoplasms of the immune system: classification by presumed cell of origin.

**DOI:** 10.1038/bjc.1975.219

**Published:** 1975-08

**Authors:** R. D. Collins


### NEOPLASMS OF THE IMMUNE SYSTEM: CLASSIFICATION BY PRESUMED CELL OF ORIGIN

R. D. COLLINS, Vanderbilt University School of Medicine, Nashville, Tennessee.

Current classifications of malignant lymphomata were developed before the complexities of the immunological system were recognized, and are defective by not providing a conceptual approach to the relationship between lymphoid neoplasms and normal lymphocyte populations. Lukes and Collins (1974*a*, *b*) have recently outlined a classification of lymphoid neoplasms based on modern concepts of the immune system, as well as histological features (Table I). The basic tenet of this proposal is that malignant lymphomata, as neoplasms of the immune system, should be classified according to the kind of lymphocyte from which they arise. Although the clinical usefulness of this proposed classification has not been tested, preliminary studies indicate that most lymphoid neoplasms may be satisfactorily categorized by a combination of functional and structural investigations.

For example, in a recent study at Vanderbilt (Leech *et al.*, 1974; Glick *et al.*, 1974), cells

TABLE—*Classification of Malignant Lymphomata (Modified from Lukes and Collins 1974a, b)*

- I. Undefined cell type
- II. T-cell types
  1. Thymic lymphoma (convoluted lymphocytic)
  2. Node based T cell lymphoma
  3. Mycosis fungoides and Sezary's syndrome
  4. T cell ALL and CLL
  5. ? Immunoblastic sarcoma (of T cells)
  6. ? Hodgkin's disease
- III. B-cell types
  1. Small lymphocyte (CLL)
  2. Plasmacytoid lymphocytic
  3. Follicular-centre cell (FCC) types
     - (a) small cleaved
     - (b) large cleaved
     - (c) small transformed—Burkitt's lymphoma
     - (d) large transformed
  4. Immunoblastic sarcoma (of B cells)
- IV. Histiocytic types
- V. Unclassifiable.

from lymphomata containing neoplastic follicular structures with one exception had surface Ig, and closely resembled follicular centre cells (FCC) ultrastructurally. Similar findings were described in those diffuse lymphomata containing, by light microscopy, cleaved FCC or a mixture of cleaved and transformed FCC. FCC lymphomata made up 58% of the 45 consecutive lymphomata studied. These studies further showed that many lymphomata previously diagnosed as "histiocytic" lymphoma were composed of cells which bore surface Ig, did not have the cytochemical reactions of histiocytes, and were similar to transformed lymphocytes by ultrastructural examination. A complex group of neoplasms of T cell origin, some arising in lymph nodes, was found.

On the basis of these and similar studies of a small number of cases, it seems likely that each sub-population of lymphoid cells may give rise to a distinct neoplasm, and that lymphoid neoplasms may be usefully classified by a combined functional and structural evaluation (Table I). The use of such classifications will significantly improve interchange of information about normal lymphoid populations with information about lymphoid neoplasms. By this approach, relationships between diseases with altered immunological functions and lymphoid malignancies will be more apparent, relationships between lymphoid neoplasms primarily affecting the marrow and lymphoid neoplasms primarily involving the extramarrow tissues may be established, and perhaps most importantly, lymphoid neoplasms may be used more effectively to study the normal lymphoid apparatus.

REFERENCES

GLICK, A. D., LEECH, J. H., WALDRON, J. A., FLEXNER, J. M., HORN, R. G. & COLLINS, R. D. (1974) Malignant Lymphomas of Follicular Centre Cell Origin. II. Ultrastructural and Cytochemical studies. *J. natn. Cancer Inst.*

LEECH, J. H., GLICK, A. D., WALDRON, J. A., FLEXNER, J. M., HORN, R. G. & COLLINS, R. D. (1974) Malignant Lymphomas of Follicular Centre Cell Origin. I. Immunologic studies. *J. natn. Cancer Inst.*

LUKES, R. J. & COLLINS, R. D. (1974*a*) A Functional Approach to the Classification of Malignant Lymphoma. In *Recent Results in Cancer Research*, Diagnosis and Therapy of Malignant Lymphoma. Ed. K. Musshoff. Heidelberg: Springer, **46**, 18.

LUKES, R. J. & COLLINS R. D. (1974*b*) Immunologic Characterization of Human Malignant Lymphomas. *Cancer N.Y.*, **34**, 1488.